# Gut microbiota dysbiosis in infantile cholestatic hepatopathy

**DOI:** 10.3389/fped.2025.1547958

**Published:** 2025-03-24

**Authors:** Yi Zou, Wenhao Ni, Yong Zhou, Dan Sun, Feng Chen, Xianyun Li

**Affiliations:** ^1^ Department of Clinical Laboratory, The Central Hospital of Enshi Tujia and Miao Autonomous Prefecture, Enshi, China; ^2^ Department of Technology, Puluo (Wuhan) Medical Biotechnology Co., Ltd., Wuhan, China; ^3^ Department of Marketing, Wuhan Kindstar Clinical Diagnostic Institute Co., Ltd., Wuhan, China; ^4^ Department of Neurology, Wuhan Children’s Hospital of Tongji Medical College, Huazhong University of Science and Technology, Wuhan, China; ^5^ Emergency and Critical Care Medical Center, Wuhan Children’s Hospital of Tongji Medical College, Huazhong University of Science and Technology, Wuhan, China

**Keywords:** infantile cholestatic hepatopathy, intestinal microbiota, 16S rDNA, *Streptococcus*, biomarker

## Abstract

**Background:**

Cholestatic hepatopathy is common in infants. While many studies link gut microbiota to liver and gallbladder diseases, the relationship between infantile cholestatic hepatopathy (ICH) and gut microbiota remains unclear.

**Methods:**

We collected stool samples from 19 healthy controls and 33 infants with ICH aged ≤3 months, then determined the intestinal microbiota by 16S rDNA sequencing. The differences of microbiota structure and functional between the two groups were analyzed.

**Results:**

Alpha-diversity analysis showed that the Chao1 and ACE indexes were significantly higher in the ICH group than control group (*p* < 0.05). LEfSe analysis showed that 18 bacteria taxa, including *Streptococcus, Streptococcaceae,* and *Staphylococcales,* enriched significantly in the ICH group, and 3 bacteria taxa were enriched in the control group. At the genus level, the relative abundance of *Streptococcus, Escherichia-Shigella,* and *Lactobacillus* in ICH group was higher than control group (*p* < 0.05). The Receiver Operating Characteristic (ROC) analysis demonstrated that *Streptococcus* was highly valuable in distinguishing ICH from healthy controls. Moreover, functional prediction analysis identified 59 metabolic pathways potentially associated with ICH.

**Conclusion:**

Gut microbiota dysbiosis is associated with infantile cholestatic hepatopathy, and *Streptococcus* can be used as an essential biomarker to identify ICH.

## 1 Introduction

Infantile cholestatic hepatopathy (ICH) is a group of hepatobiliary diseases that occur within the first 3 months of life (1). It is primarily manifested by yellowish staining of the skin, darkened urine color, and lightened stool color, among other symptoms, gradually progressing to hyperbilirubinemia. It may even result in cirrhosis and liver failure, ultimately causing death. Currently, ICH is a leading cause of death and disability during infancy. The etiology of infantile cholestasis is complex and mainly includes obstructive causes (such as biliary atresia), infections (such as hepatitis viral infection), endocrine disorders (such as hypothyroidism), drug poisoning (such as parenteral nutrition-related liver disease), genetics (such as familial intrahepatic cholestasis and congenital bile acid synthesis defects), metabolism (such as abnormal amino acid metabolism and abnormal lipid metabolism), idiopathic infantile cholestasis, and others (2). However, cholestasis caused by different reasons is clinically manifested as abnormal bilirubin levels, so what exactly causes this phenomenon?

Research studies have demonstrated that there is a tight affiliation between the composition of the gut microbiota and serum bilirubin levels (3). The gut microbiota participates in the metabolic process of bilirubin. Dysbiosis may impede bilirubin metabolism, leading to intestinal bilirubin accumulation and contributing to hyperbilirubinemia. Notably, specific gut microbiota, including *Clostridium, Bifidobacterium*, and members of the *Firmicutes* can convert bilirubin into metabolites that are prone to excretion through specific enzymatic reactions, thereby maintaining the homeostasis of bilirubin within the body (4). Furthermore, an imbalance in the gut microbiota may also lead to the disruption of the intestinal mucosal barrier, augmenting the likelihood of bilirubin entering the blood circulation via the intestinal mucosa and further exacerbating the accumulation of bilirubin (5). In pathologies such as neonatal jaundice, dysbiosis of the gut microbiota are also regarded as one of the crucial factors contributing to elevated bilirubin levels. Studies have ascertained that maternal supplementation with probiotics during pregnancy can facilitate the metabolism of neonatal bilirubin, lower neonatal bilirubin levels, and thereby reduce the incidence of neonatal jaundice (6). These research findings imply that high bilirubin in ICH is associated with abnormal gut microbiota, yet the role of gut microbiota in ICH still demands further exploration.

This study utilized 16S rDNA sequencing to compare gut microbiota profiles between healthy infants and ICH patients, aiming to identify microbial biomarkers linked to ICH pathogenesis thereby further exploring the potential role through which the gut microbiota induces ICH.

## 2 Materials and methods

### 2.1 Research subjects

Healthy control individuals and infants with ICH were enrolled from The Central Hospital of Enshi Tujia and Miao Autonomous Prefecture (Enshi, China) spanning from June 2021 to December 2023. The diagnostic criteria for infants with cholestatic hepatopathy were in accordance with the regulations stipulated in the guidelines for the assessment of infantile cholestatic hepatopathy of the North American Society for Pediatric Gastroenterology, Hepatology and Nutrition (7, 8). The infants with cholestatic hepatopathy in this study satisfied the following inclusion criteria: (1) Infants aged less than 3 months; (2) All diagnostic criteria for cholestasis were met at admission; (3) No antibiotics or probiotics were administered during the preceding 2 weeks; The control group comprised infants aged less than 3 months with normal health as indicated by physical examinations in the Department of Pediatrics; infants with no comorbidities (e.g., cholestasis or congenital developmental abnormalities) no antibiotics or probiotics were administered in the preceding 2 weeks. This study complied with the Declaration of Helsinki. It has been meticulously examined and approved by the Human Ethics Committee of The Central Hospital of Enshi Tujia and Miao Autonomous Prefecture (Approval Number: 2024-084-01). Moreover, we have ensured the attainment of explicit and written informed consent from the parents of each child involved in the research.

### 2.2 Clinical information and stool samples collection

We recorded the age of each inclusion population stool samples were collected from patients in the ICH group within the first 3 days after admission. Meanwhile, the stool samples of the control group were obtained from healthy individuals undergoing physical examinations. Each fecal sample weighed approximately 10 g. All samples were stored at −80°C immediately after collection and subsequently processed for batch analysis.

### 2.3 DNA extraction, library construction and sequencing

Genomic DNA was extracted using Omega E.Z.N.A. Stool DNA Kit (MoBio Laboratories, USA) per manufacturer's protocol. Subsequently, the quality of the extracted DNA samples was inspected in 1% agarose gel electrophoresis. The V3-V4 regions of 16S rDNA were amplified with primers 338F (5′-ACTCCTACGGGAGGC AGCA-3′) and 806R (5′-GGACTACHVGGGTWTCTAAT-3′). PCR products were separated by gel electrophoresis, purified by E.Z.N.A.® Gel Extraction Kit (Omega, USA), quantified by NanoDrop 2000 (Thermo Scientific, USA), and then used for library preparation with the NEB Next® Ultra™ II DNA Library Prep Kit (New England Biolabs, USA). Finally, sequencing was performed on Illumina MiSeq PE250 platform (Illumina, San Diego, CA, USA).

### 2.4 Taxonomical annotation

Raw data were filtered to remove sequences less than 200 bp in length, and chimeric sequences were removed by comparing with the Gold Database using the UCHIME method (9). High-quality sequences were processed for Amplicon Sequence Variants (ASV) determination using the VSEARCH (v2.7.1) software and the DADA2 algorithm (v1.16), with a sequence similarity threshold of 100% (10). Comparison with the Silva 132 rRNA gene database using the RDP Classifier algorithm was performed, and a confidence threshold of 80% was set to obtain the species classification information corresponding to each ASV.

### 2.5 Statistical analysis

SPSS (v23.0) (IBM Corp., USA) was used for statistical analysis of infant ages. Based on species annotation and relative abundance results, the Venn diagram visualized by QIIME2 was employed to exhibit the richness and similarity of the gut microbiota composition among the groups, the species composition and the cluster analysis of species abundance were performed using R Studio R(v3.5.1) software (11). The alpha-diversity analysis (including Shannon, Chao1, ACE and Simpson indices) was performed using QIIME2 (v2020.11.0) software. The beta-diversity analysis (Bray-Curtis distance) was performed basing on standardized ASV abundance tables to evaluate the community composition and structure of gut microbiota. Linear discriminant analysis Effect Size (LEfSe) analysis was employed to identify the features that contributed the most to the variation between the control and ICH groups. The disparities in the relative abundance of gut microbiota at phylum and genus levels were exploited to assess the differences in gut microbiota composition between the control and ICH groups. The Receiver Operating Characteristic (ROC) curve calculated and displayed by R software (v4.1.3) was utilized to evaluate effective biomarker for ICH. Predictive functional analysis (PICRUSt) was utilized to predict the functional composition of the microbial community based on its 16S profile. The KEGG database was utilized to acquire KO pathway. The STAMP (v2.1.3) software package was exploited for analyzing KEGG pathway profiles (12).

## 3 Results

### 3.1 Characteristics of population

A total of 52 infants aged no more than 3 months were included in this study, among whom 19 infants (10 males and 9 females) were in the healthy control group, with a mean age of 2.02 ± 0.65 months; within the ICH group, 33 infants (17 males and 16 females) were enrolled, with a mean age of 1.74 ± 0.69 months. The feeding patterns in the healthy control group were as follows: 10 infants were breastfed, 3 were formula-fed, and 6 were mixed-fed; in the ICH group, 15 infants were breastfed, 5 were formula-fed, and 11 were mixed-fed. The etiologies of ICH included cholestatic hepatitis (*n* = 20), cholestatic hepatitis with cytomegalovirus infection (*n* = 3), biliary atresia (*n* = 4), metabolic genetic syndromes (*n* = 5), and progressive familial intrahepatic cholestasis type 2 (*n* = 1). No significant differences in age, sex, or feeding patterns were observed (*p* > 0.05).

### 3.2 Clusters in infant gut microbiota

**Figure 1:**
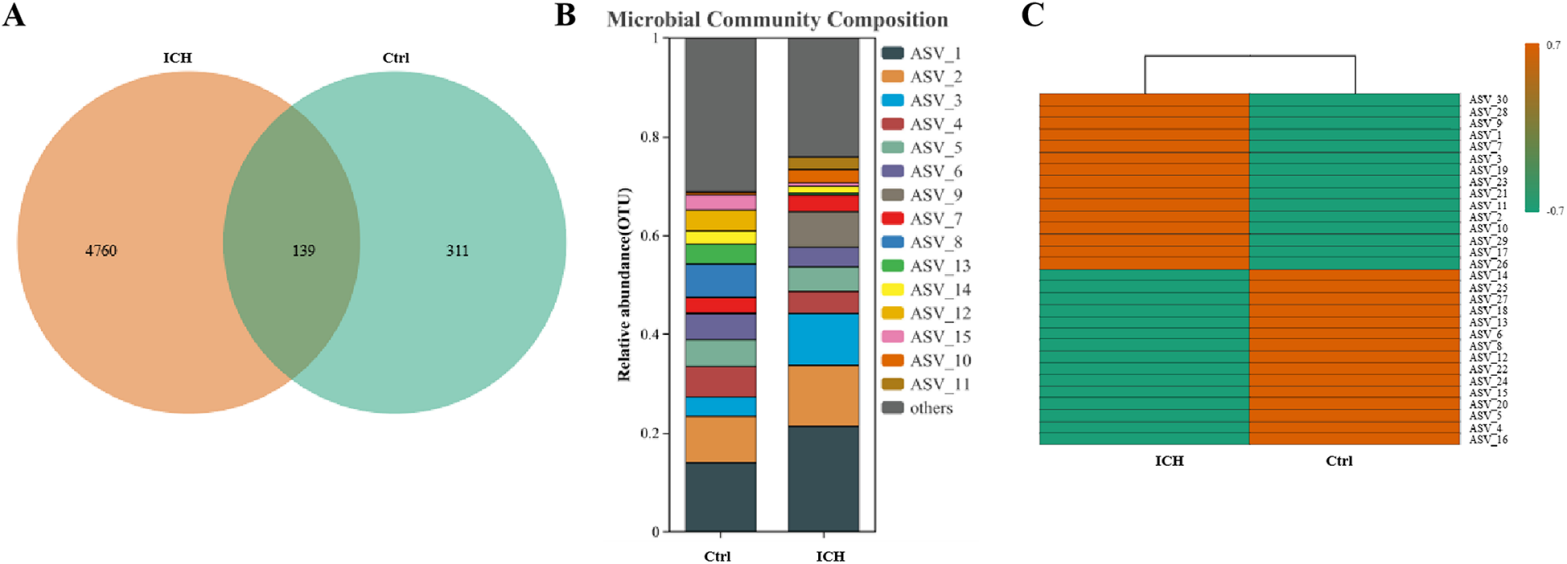
Diversity analysis of gut microbiota between the control group and the ICH group. **(A)** Venn diagram of ASVs among the two groups. **(B)** Relative abundance of ASV among the two groups. **(C)** Cluster analysis of species abundance among the two groups. Ctrl, control group; ICH, infantile cholestatic hepatopathy group; ASV, amplicon sequence variants.

This 16S rDNA sequencing generated 83,983 ± 23,676 reads per sample. Cluster analysis identified 5,210 ASVs, with 139 shared between groups, 4,760 unique to ICH, and 311 unique to controls (Figure 1A). Sequencing data disclosed significant differences in the relative expression levels of ASVs between the healthy control group and the ICH group (Figure 1B). The depth of the orange color in the figure represents the abundance level of each ASV among the group, the more intense the orange hue is, the relatively higher the abundance of the ASV. The results indicated that ASV_30, ASV_28, and ASV_9, among others, had a higher abundance in the ICH group, whereas ASV_14, ASV_25, and ASV_27 showed a higher abundance in the control group (Figure 1C).

### 3.3 Fecal alpha and beta diversity

**Figure 2:**
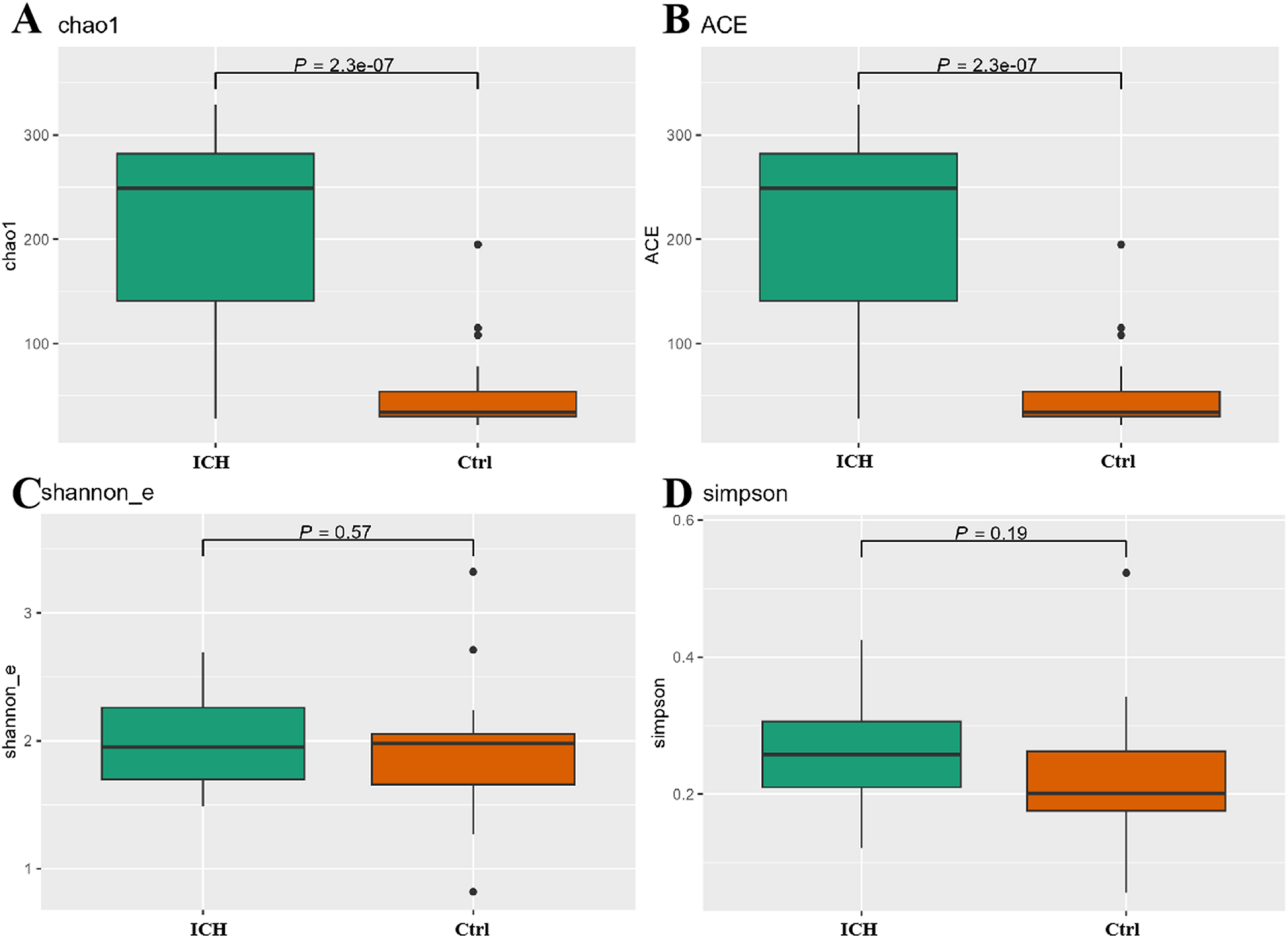
Differences of *α*-diversity between the control group and the ICH group. **(A)** Chao1 index. **(B)** ACE index. **(C)** Shannon_e index. **(D)** Simpson index. Ctrl, control group; ICH, infantile cholestatic hepatopathy group.

Alpha-diversity analysis showed that the Chao1 and ACE indexes were significantly higher in ICH (*p* < 0.05) (Figure 2A), whereas Shannon index and Simpson index showed no significant differences (*p >* 0.05) (Figure 2B). Beta-diversity analyses (Bray-Curtis distance, NMDS1, PCA, PCOA) revealed no group separation (*p >* 0.05) (Supplementary Figures S1A–D).

### 3.4 Lefse analysis

**Figure 3:**
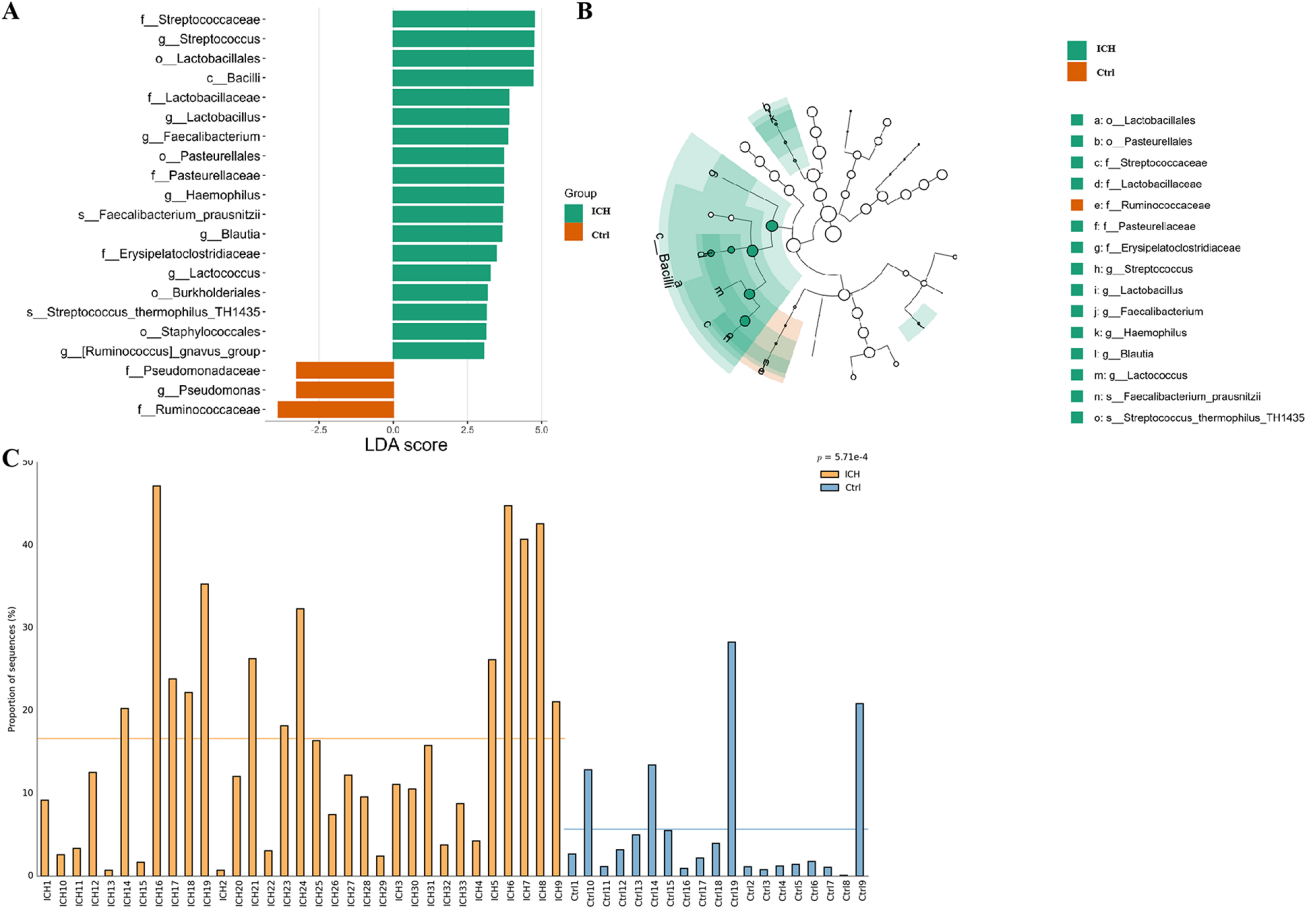
LEfSe analysis results of the control group and the ICH group. **(A)** Bar plot of the LDA Score between two groups. LDA scores on the log 10 scale are indicated at the bottom. **(B)** Evolutionary branching diagram of LEfSe analysis based on taxonomic information between groups. **(C)** Histogram of the *Streptococcaceae* relative abundances between groups. Ctrl, control group; ICH, infantile cholestatic hepatopathy group; LDA, linear discriminant analysis; LEfSe analysis, linear discriminant analysis effect size analysis.

Based on the threshold of Linear discriminant analysis (LDA) >3.0, it was ascertained that there existed significant disparities in 21 features between the control group and the ICH group. Of those, 18 bacteria taxa (*o, f*, and *g*, respectively, representing order, family, and genus level) were more prevalent in the ICH group, while 3 bacteria taxa were more prevalent in the control group (Figure 3A). From the LEfSe cladogram, we also found that these important bacteria were significantly abundant in the different groups (Figure 3B). Additionally, it was found that the relative abundance of *Streptococcaceae* in ICH samples was significantly higher than that in healthy controls (Figure 3C).

### 3.5 Difference expression analysis of gut microbiota between the ICH and control groups

We also explored in the gutMDisorder database whether the aforementioned gut microbiota related to ICH exhibited the same alteration patterns in other diseases (13), and the outcomes are presented in Table 1, or the opposite pattern of change with intervention measures, and the outcomes showed that the increase of *Streptococcaceae* and *Streptococcus* were correlated with Crohn's disease, and the reduced abundance was observed by intervention with Infliximab.

**Table 1:** The gut microbes associated with ICH have the same pattern of change as other diseases searched in the gutMDisoeder database.

Gut microbe	Altertion	Disorder	PMID
Streptococcaceae	UP	Colorectal Neoplasms	28153960
	UP	Hospital admission; Anorexia Nervosa	26428446
	UP	Acute viral gastroenteritis without complication	28397879
Streptococcus	Up	Diarrhea	24803517
	Up	Diabetes Mellitus; Type 2	27151248
	Up	B. bifidum PRL2010; Colitis; Ulcerative	27604252
	Up	Depressive Disorder; Major	27741466
Lactobacillales	Up	Autoimmune liver disease	29969462
Bacilli	Up	Colitis; Ulcerative	22170749
	Up	Colorectal conventional adenoma	28038683
Lactobacillaceae	Up	Lactobacillus casei	29264969
Lactobacillus	Up	Colitis; Ulcerative	17897884
	Up	Crohn Disease	17897884
	Up	Irritable Bowel Syndrome	21330412
	Up	Colorectal Neoplasms	22622349
Faecalibacterium	Up	Hospital admission; Anorexia Nervosa	26428446
	Up	Hepatolenticular Degeneration	30075590
Pasteurellaceae	Up	First-degree relatives of children with Crohn’s disease	28222161
Haemophilus	Up	Pediatric ulcerative colitis	27217061
	Up	Arthritis; Rheumatoid	34721377
Faecalibacterium prausnitzii	Up	Colitis;Ulcerative	17265126
	Up	Obesity	19849869/24292154
	Up	Dermatitis; Atopic	26431583
	Up	Eczema	27812181
Blautia	Up	Diarrhea	24803517
	Up	Constipation	25073603
	Up	Hospital admission; Anorexia Nervosa	26428446
	Up	Cholangitis; Sclerosing	26857969
	Up	Diabetes Mellitus; Type 1	27231166
	Up	Parkinson Disease; Cholinergic Antagonists	28195358
	Up	Non-alcoholic Fatty Liver Disease	28823367/29948900
	Up	Obesity	29214176
	Up	Diarrhea	24803517
	Up	Constipation	25073603
Ruminococcus gnavus	Up	Colorectal Neoplasms	22622349
	Up	Colitis; Ulcerative	20648002
	Up	Crohn Disease; No Inflammation/Inflammation	20648002
	Up	Eczema	27812181
	Up	Colitis; Ulcerative	28039159
Pseudomonas	Down	Colorectal Neoplasms	28600626
	Down	Pancreatic head carcinoma	29653723
	Down	Ulcerative colitis at exacerbated stage	29796862
	Down	Depressive Disorder; Major	33421868

**Figure 4:**
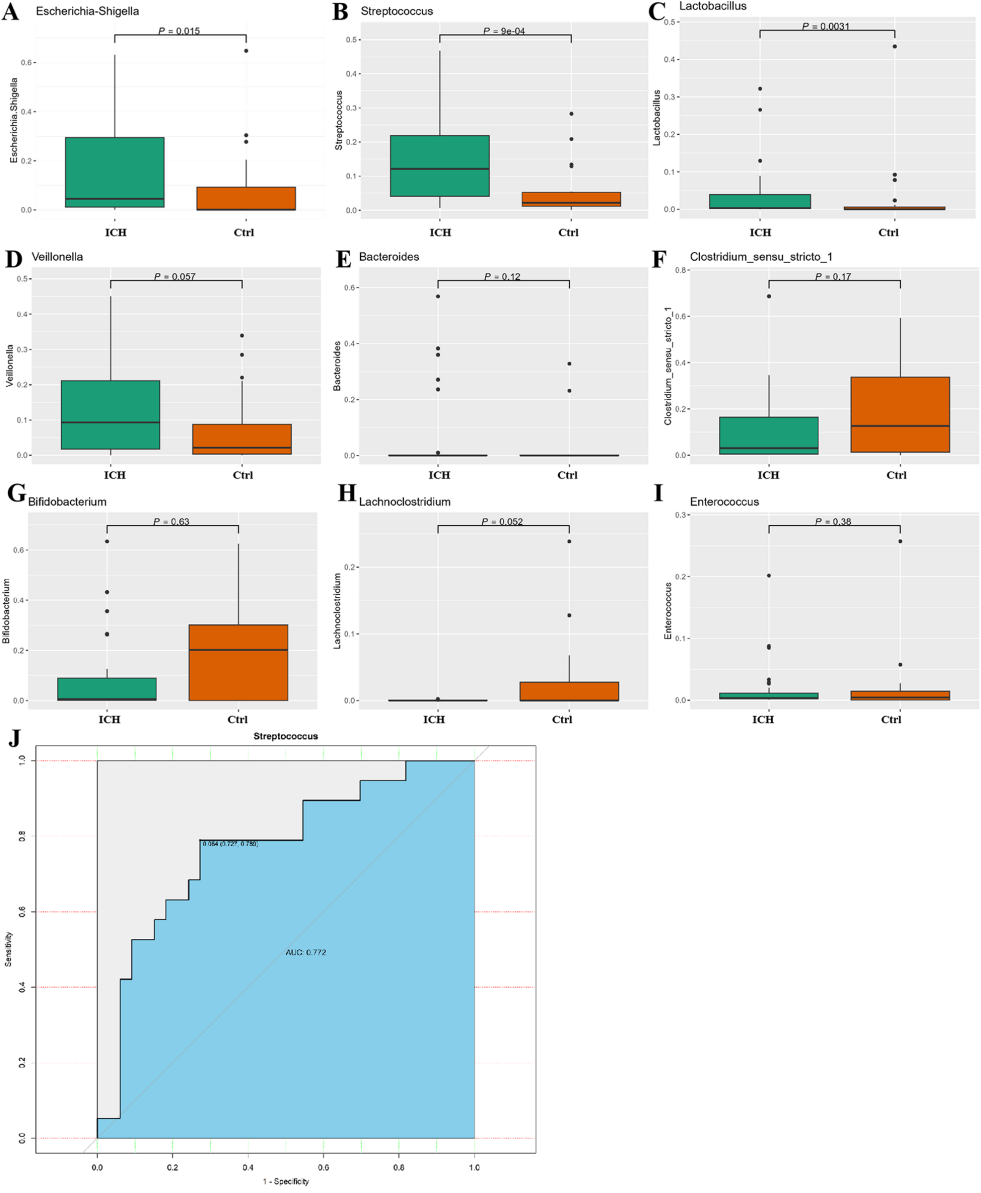
Difference relative expression of gut microbiota between the control group and the ICH group. **(A–I)** Differences in relative abundance between control group and ICH group at the genus level. **(J)** ROC curve of *steptococcus, Lactobacillus* and *Escherichia-Shigella* for distinguishing ICH. Ctrl, control group; ICH, infantile cholestatic hepatopathy group; ROC, receiver operating characteristic.

We mainly compared the differences of taxa at the phylum and genus levels. At the phylum level (Supplementary Figure S2), compared with the control group, the relative abundance of *Actinobacteriota* and *Bacteroidota* increased in the ICH group, while the relative abundance of *Firmicutes* and *Proteobacteria* decreased, but there were no statistical differences (*p* > 0.05). At the genus level, compared with the control group, the relative abundance of *Escherichia-Shigella*, *Streptococcus,* and *Lactobacillus* was significantly elevated in the ICH group (*p* < 0.05) (Figures 4A–C). The abundance of *Veillonella* and *Bacteroides* increased in the ICH group (Figures 4D–E), while the relative abundance of *Clostridium_sensu_stricto_1*, *Bifidobacterium*, *Lachnoclostridium,* and *Enterococcus* was lower in ICH group (Figures 4F–I), but the differences were not statistically significant (*p* *>* 0.05). The ROC curve was used to evaluate the effective biomarkers of ICH. The results showed that *Streptococcus* [Area Under the ROC Curve (AUC) = 0.772, clearly distinguish ICH from healthy control] was the effective biomarker of ICH (Figure 4J). Additionally, the AUC values of *Lactobacillus* and *Escherichia-Shigella* were 0.748 and 0.705, respectively (Supplementary Figure S3).

### 3.6 Predictive functional analysis

**Figure 5:**
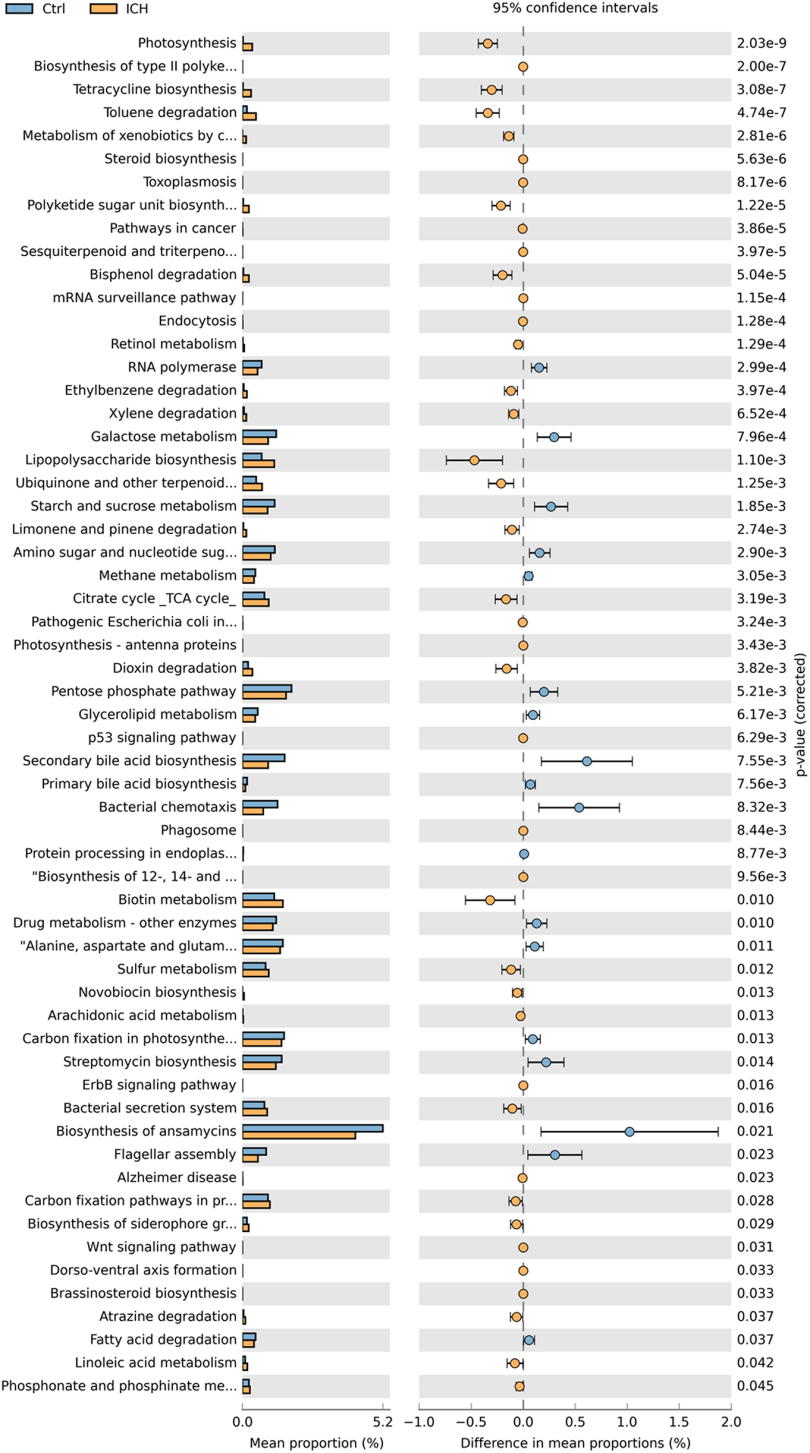
The KEGG orthologs predicted to be related to the occurrence of ICH. Ctrl, control group; ICH, infantile cholestatic hepatopathy group.

PICRUSt functional analysis predicted that 59 metabolic pathways associated with ICH, including Biosynthesis, Metabolism, and Signal pathway (Figure 5). Compared with the control group, the ICH group exhibited increased Lipopolysaccharide biosynthesis and Biotin metabolism, alongside reduced Secondary bile acid biosynthesis, Bacterial chemotaxis, Biosynthesis of ansamycins, and Flagellar assembly processes.

## 4 Discussion

The enterohepatic circulation, serving as a crucial link in the pathogenesis of cholestatic liver diseases, has garnered extensive scientific attention. Among these, alterations in the intestinal microbiota are regarded as a potential element in the pathogenesis of ICH. This study analyzed the composition and changes of gut microbiota in healthy control group and ICH group using high-throughput 16S rDNA sequencing technology. The research findings indicate that there are substantial differences in the composition of the intestinal microbiota between ICH patients and healthy controls, while alpha-diversity indices (Chao1 and ACE) were significantly elevated in the ICH group. Additionally, both the ICH group and the control group possess unique characteristic bacterial strains, and *Streptococcus* could be used as an essential biomarker to identify ICH. This discovery offers new leads for us to further comprehend the pathogenesis of ICH.

Alpha-diversity provides indispensable statistical information for microbial communities, and its high-level index highlights the traits of species diversity and even distribution within the community. In this study, alpha-diversity indices (Chao1 and ACE) were significantly elevated in the ICH group, reflecting increased microbial richness. However, no significant differences were observed in Shannon and Simpson indices, suggesting comparable evenness between groups. The species richness of gut microbiota in infants with ICH has considerably increased, and this alteration may stem from the increase of potentially pathogenic species (14). Contrary to our findings, studies on primary biliary cholangitis (PBC) and cirrhosis reported reduced alpha-diversity indices (e.g., Shannon and Simpson) and depletion of commensal taxa such as *Faecalibacterium* and enrichment of pathogenic *Enterobacteriaceae* (15). Similarly, cirrhotic patients exhibited decreased beneficial *Bifidobacterium* and increased pro-inflammatory *Escherichia/Shigella* species, correlating with disease severity (16, 17). Liu et al. (18) further confirmed through a meta-analysis that gut microbiota diversity inversely correlates with Child-Pugh scores in cirrhosis, highlighting the ecological collapse of symbiotic networks. The significant difference in alpha-diversity between the two groups sets the stage for the next search for pathogenic strains of ICH.

Beta-diversity analysis revealed a high similarity of the intestinal microbiota between the two groups in our study. Dong et al. (19) employing 16S rRNA gene sequencing technology, discovered that there was no significant disparity in the beta-diversity of the intestinal microbiota between jaundiced children and non-jaundiced children. Likewise, Zheng et al. (20) disclosed, via 16S rDNA gene sequencing, the non-difference in the beta-diversity of the intestinal microbiota between children with pathological jaundice and those with physiological jaundice. This finding might imply that the beta-diversity of the intestinal microbiota is not directly associated with the cholestatic.

In our research, no significant differences in abundance were demonstrated between the two groups at the phylum level; however, at the genus level, the ICH group exhibited significantly increased *Streptococcus* and *Escherichia-Shigella* abundance, alongside reduced *Lactobacillus* levels (*p* < 0.05). Additionally, LEfSe analysis further confirmed that *Streptococcus* as the dominant taxon in ICH. It is notable that the increase of *Streptococcus* in stool samples of infants with cholestasis is not mentioned for the first time. Zhang et al. (21) discovered through 16S rRNA gene sequencing technology that compared with healthy infants, *Clostridium, Gemella, Streptococcus, Veillonella*, and *Enterobacteriaceae* were significantly enriched in the ICH group, and these alterations were positively correlated with serological indicators of impaired liver function. The study by Zhong et al. (22) also disclosed similar results through 16S rRNA gene sequencing, they found that the quantities of *Veillonella, Streptococcus*, and *Clostridium spp* in the intestinal microbiota of the infant cholestasis group were higher compared to the healthy infant group (*p* < 0.05). The relationship between the *Streptococcus* and cholestasis, especially certain species of *Streptococcus*, such as *beta-hemolytic Streptococcus*, may lead to biliary duct infections. The toxins and metabolites produced by these bacteria can stimulate the liver, which resulting in local inflammatory responses and fibrotic processes. Chronic and recurrent inflammation can cause thickening of the bile duct wall and scar formation, thereby impairing the normal excretion of bile. Therefore, the excessive presence of *Streptococcus* may pose a potential threat to the intestinal and hepatobiliary health of infants.

In addition to *Streptococcus*, the association of *Veillonella* with cholestasis (20–22), primary cholangitis and biliary cirrhosis (23, 24) has been affirmed by numerous studies. Our study also discovered that, compared with the control group, the abundance of *Veillonella* in the ICH group exhibited an ascending tendency. Nevertheless, due to the limited sample size, the disparity between the two groups did not attain statistical significance (*p* *>* 0.05). The *Veillonella* genus, being a sort of Gram-negative anaerobic micrococcus, predominantly resides in the oral cavity, intestine and respiratory tract of humans and animals. They frequently cause inflammation as a result of mixed infections, and this mixed infection may be linked to the generation of endotoxin. Given this potential pathogenicity of the *Veillonella* genus, the elevated abundance undoubtedly deserves close attention from clinicians.

The LEfSe analysis results of this study indicated that *Ruminococcus gnavus* was the predominant strain in the ICH group. Although there are no reports to date regarding the direct relationship between the abundance of *Ruminococcus gnavus* and cholestasis, the study by Lee et al. (25) disclosed that a specific subtype within this strain (such as *Ruminococcus gnavus 53*) encodes 7β-hydroxysteroid dehydrogenase (7β-HSDH) and is capable of generating Ursodeoxycholic acid (UDCA). UDCA, as a secondary bile acid under the influence of intestinal flora, has been proposed for use in patients with cholestatic liver disease (26). Nevertheless, the results of this study revealed that, in comparison with the healthy control group, the abundance of the *Ruminococcus gnavus_group* in the ICH group increased significantly. We hypothesize that this might be closely related to the body's intrinsic immune regulatory mechanism. Additionally, although previous studies have substantiated that the purified *Ruminococcus gnavus 53* has the property of generating UDCA, the *Ruminococcus gnavus_group* involved in this study might have variations at the species level. In light of the current technical constraints, we are still unable to precisely identify the specific types of bacteria within the *Ruminococcus gnavus_group*. Therefore, we will closely monitor to the development of related technologies and conduct in-depth explorations of the potential role of *Ruminococcus gnavus_group* in ICH patients, with the aim of providing novel ideas and approaches for future treatment.

In addition to Ruminococcus gnavus, other significantly enriched bacteria, such as Streptococcus and Escherichia-Shigella, may also play critical roles in the pathogenesis of ICH. For instance, *Streptococcus* has been implicated in biliary tract infections and inflammation, which can lead to bile duct obstruction and impaired bile flow (21, 22). The toxins and metabolites produced by Streptococcus may further exacerbate hepatic inflammation and fibrosis, contributing to the progression of cholestasis. Similarly, *Escherichia-Shigella*, known for its role in gut inflammation, may disrupt the intestinal barrier, leading to increased translocation of bacterial products and subsequent hepatic inflammation (23, 24). These findings suggest that the enrichment of these bacteria in ICH infants could significantly influence the disease's pathogenesis through multiple mechanisms, including inflammation, bile acid metabolism, and immune regulation.

Functional prediction analysis identified 59 metabolic pathways potentially closely related to ICH. Among these pathways, the synthesis of ansamycin showed pronounced effects on the ICH group. Ansamycin, being a helical piperidine derivative of rifamycin (27), holds a prominent position in the medical domain. It is primarily utilized for the treatment of pulmonary mycobacterial infections, exhibits antibacterial potency approximately fourfold higher than that of rifampicin and is also efficacious against tuberculosis strains resistant to rifampicin. Its adverse reactions are comparable to those of rifampicin, and the exposure is positively correlated with bilirubin concentration as well (28). However, such antibiotics would significantly inhibit *Streptococcus*, which is inconsistent with this expectation and is worth for further exploration (29). In addition, secondary bile acid biosynthesis and lipopolysaccharide biosynthesis emerged as key pathways with significant implications for disease pathogenesis. The marked reduction in secondary bile acid biosynthesis (*p* < 0.05) suggests impaired microbial conversion of primary bile acids to secondary forms (e.g., deoxycholic acid and lithocholic acid), which may lead to the accumulation of cytotoxic primary bile acids in the liver, exacerbating cholestatic injury (30). Conversely, the upregulation of lipopolysaccharide (LPS) biosynthesis pathways suggest activation of the TLR4/NF-κB pathway, inducing hepatic inflammatory responses. Chronic stimulation may lead to bile duct fibrosis and impaired bile excretion (31). These findings align with previous studies demonstrating that LPS-induced inflammation disrupts bile acid transporters (e.g., BSEP and NTCP), further impairing bile acid homeostasis (5). The results predicted by this function prediction analysis undoubtedly offer valuable references for further in-depth investigations in the future and also indicate the direction for the clinical treatment of ICH.

This study has two key strengths. Firstly, this research significantly augmented the number of samples, thereby substantially enhancing the reliability of the analytical results. Secondly, aside from conducting an in-depth exploration of the variations in intestinal flora among different groups, we specifically analyzed the diseases that exhibited a similar pattern of changes to the differential strains in this study. This provides critical insights for comprehending the potential association between intestinal flora and cholestatic liver diseases. Nevertheless, this study also presents certain limitations. Firstly, this study mainly analyzes the characteristics of intestinal flora based on cross-sectional data and lacks follow-up tracking and observation of the changes in intestinal flora after treatment. Thus, causal inferences cannot be drawn from these findings. Secondly, due to the limited sample size of the ICH, subgroups such as cholestatic hepatitis with cytomegalovirus infection, biliary atresia, and metabolic genetic syndromes each had fewer than 5 cases; therefore, subgroup analysis based on ICH etiology was not performed. Looking ahead, we will endeavor to compensate for the shortcomings of the existing research by expanding the sample size, conducting in-depth studies on the intestinal microbiota of infants at multiple time points, and analyzing the characteristic gut microbiota of ICH caused by different etiologies.

## 5 Conclusion

The richness and diversity of the intestinal microbiota in the ICH group exhibited significant dissimilarities compared to those in the control group. Through LEfSe analysis and ROC curve analysis, *Streptococcus*, an essential biomarker with notable differences, was identified. The functional prediction analysis of 16S revealed that 59 metabolic pathways may be linked to the occurrence of ICH, among which the synthesis of ansamycin had a particularly prominent influence on the ICH group.

## Data Availability

The data presented in the study are deposited in the NCBI repository (https://www.ncbi.nlm.nih.gov/bioproject/PRJNA1236511/), accession number PRJNA1236511.

## Supplementary material

Supplementary Figure S1 Differences of beta-diversity between the control group and the ICH group. **(A)** NMDS1 analysis. **(B)** PCA analysis. **(C)** PCOA analysis. **(D)** Kruskal-Wallis's test. Ctrl, Control group; ICH, Infantile cholestatic hepatopathy group; NMDS1 analysis, non-metric multidimensional scaling 1 analysis; PCA analysis, Principal Component Analysis; PCOA analysis, principal co-ordinates analysis.

Supplementary Figure S2 Difference relative expression of gut microbiota between the control group and the ICH group at the phylum level. **(A)** Firmicutes. **(B)** Proteobacteria. **(C)** Actinobacteriota. **(D)** Bacteroidota. Ctrl, Control group; ICH, Infantile cholestatic hepatopathy group.

Supplementary Figure S3 ROC curve of **(A)** Lactobacillus and **(B)** Escherichia-Shigella for distinguishing ICH. ICH, Infantile cholestatic hepatopathy; ROC, Receiver Operating Characteristic.
