# Supplementary figures and images for: Gut microbiota dysbiosis in infantile cholestatic hepatopathy
Source: Front Pediatr. 2025 Mar 24;13:1547958. doi: 10.3389/fped.2025.1547958 (PMC11973382; doi:10.3389/fped.2025.1547958)

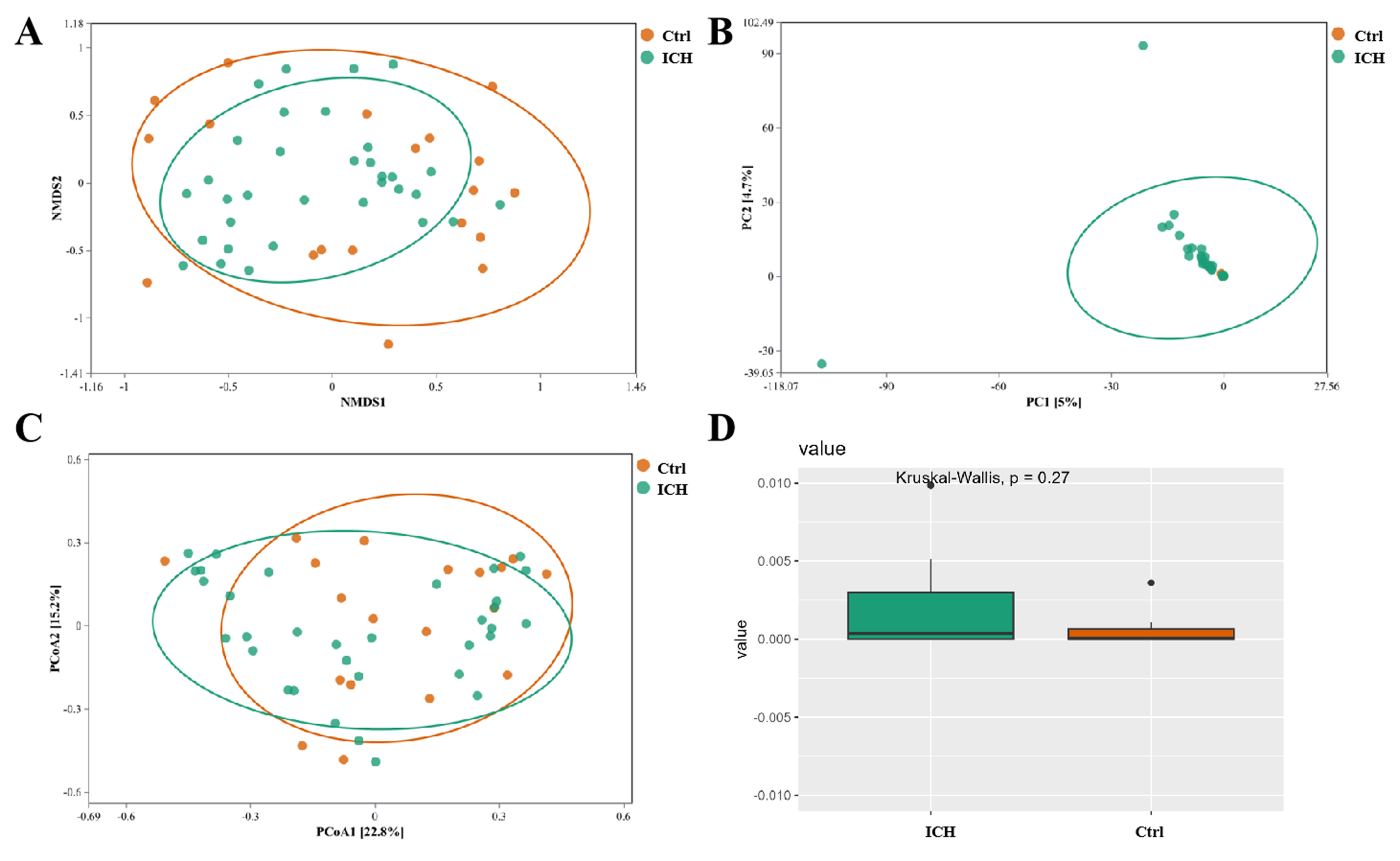

Supplement: Supplementary Figure S1 — Differences of beta-diversity between the control group and the ICH group. (A) NMDS1 analysis. (B) PCA analysis. (C) PCOA analysis. (D) Kruskal-Wallis's test. Ctrl, Control group; ICH, Infantile cholestatic hepatopathy group; NMDS1 analysis, non-metric multidimensional scaling 1 analysis; PCA analysis, Principal Component Analysis; PCOA analysis, principal co-ordinates analysis. [file Image1.tif]

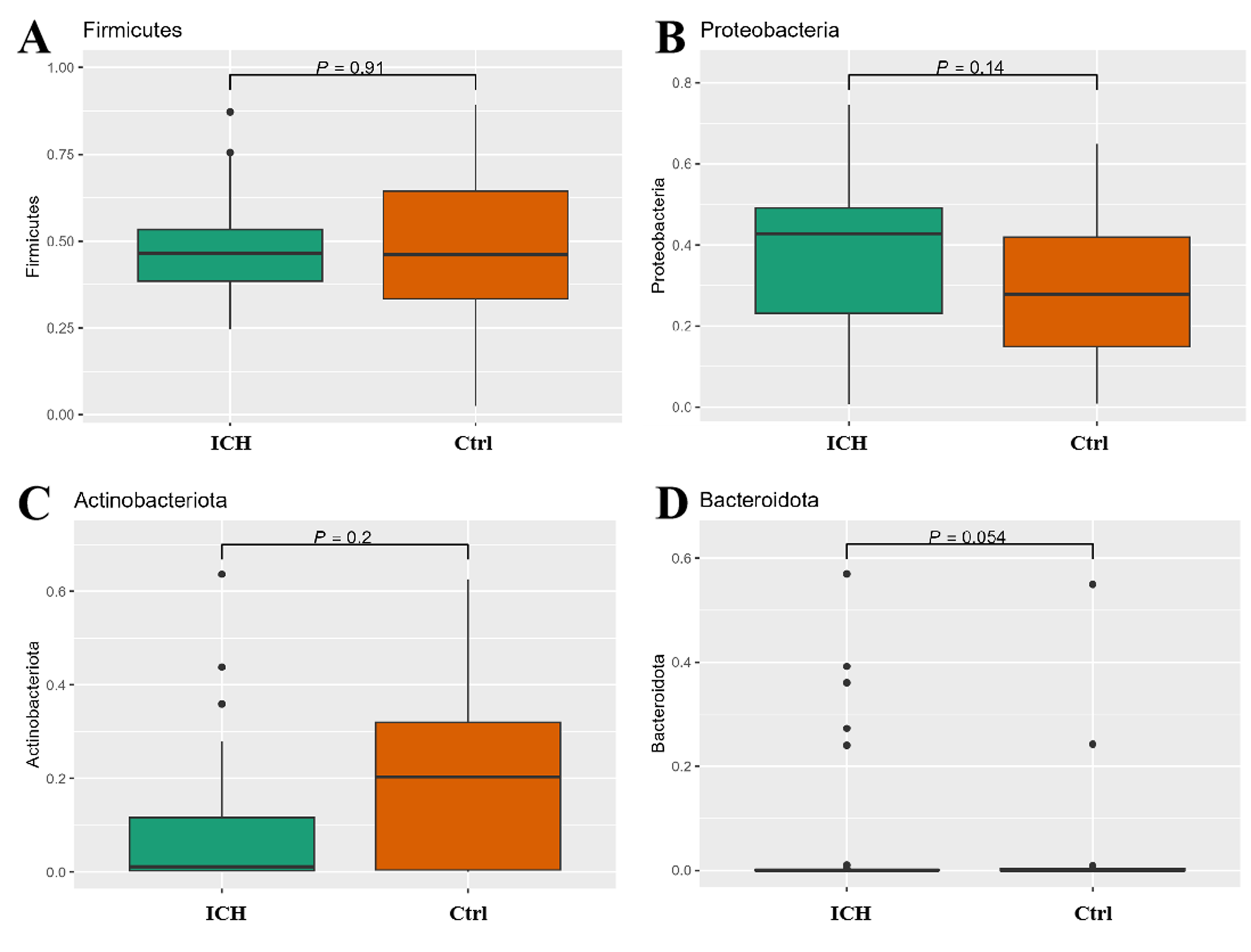

Supplement: Supplementary Figure S2 — Difference relative expression of gut microbiota between the control group and the ICH group at the phylum level. (A) Firmicutes. (B) Proteobacteria. (C) Actinobacteriota. (D) Bacteroidota. Ctrl, Control group; ICH, Infantile cholestatic hepatopathy group. [file Image2.tif]

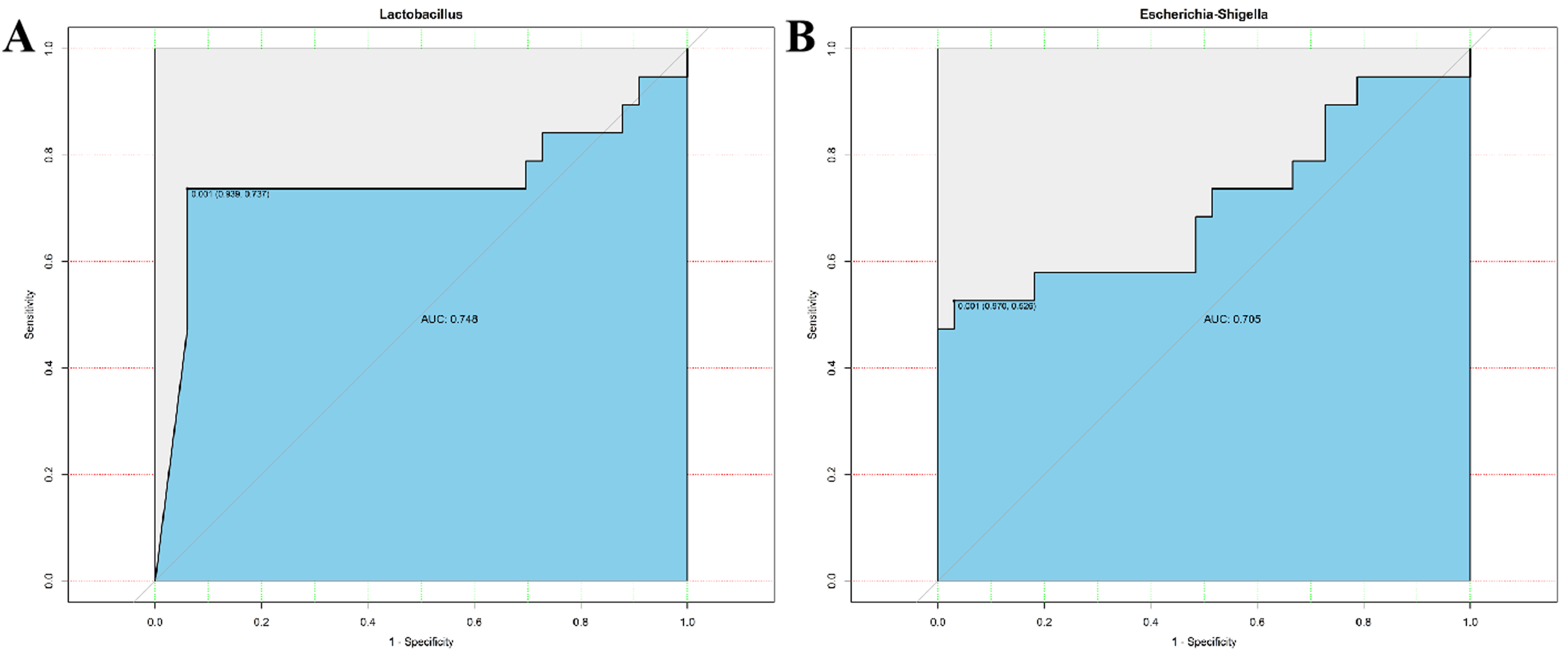

Supplement: Supplementary Figure S3 — ROC curve of (A) Lactobacillus and (B) Escherichia-Shigella for distinguishing ICH. ICH, Infantile cholestatic hepatopathy; ROC, Receiver Operating Characteristic. [file Image3.tif]
